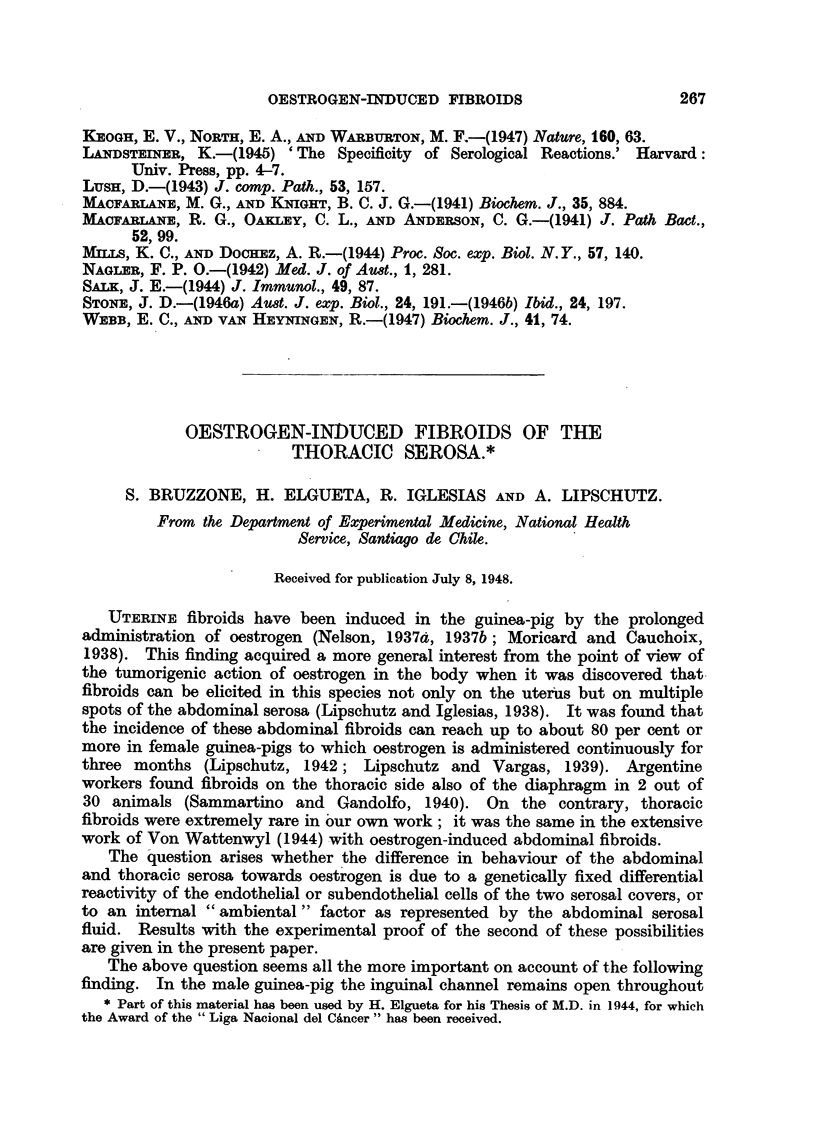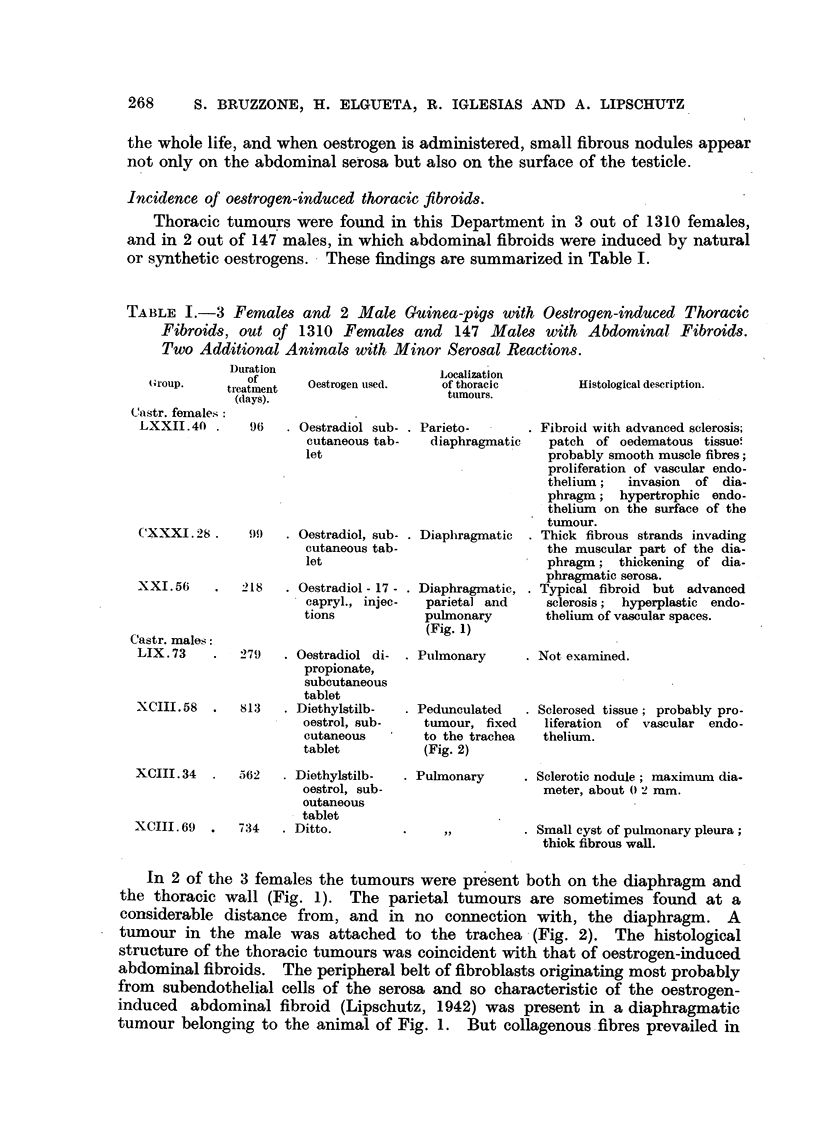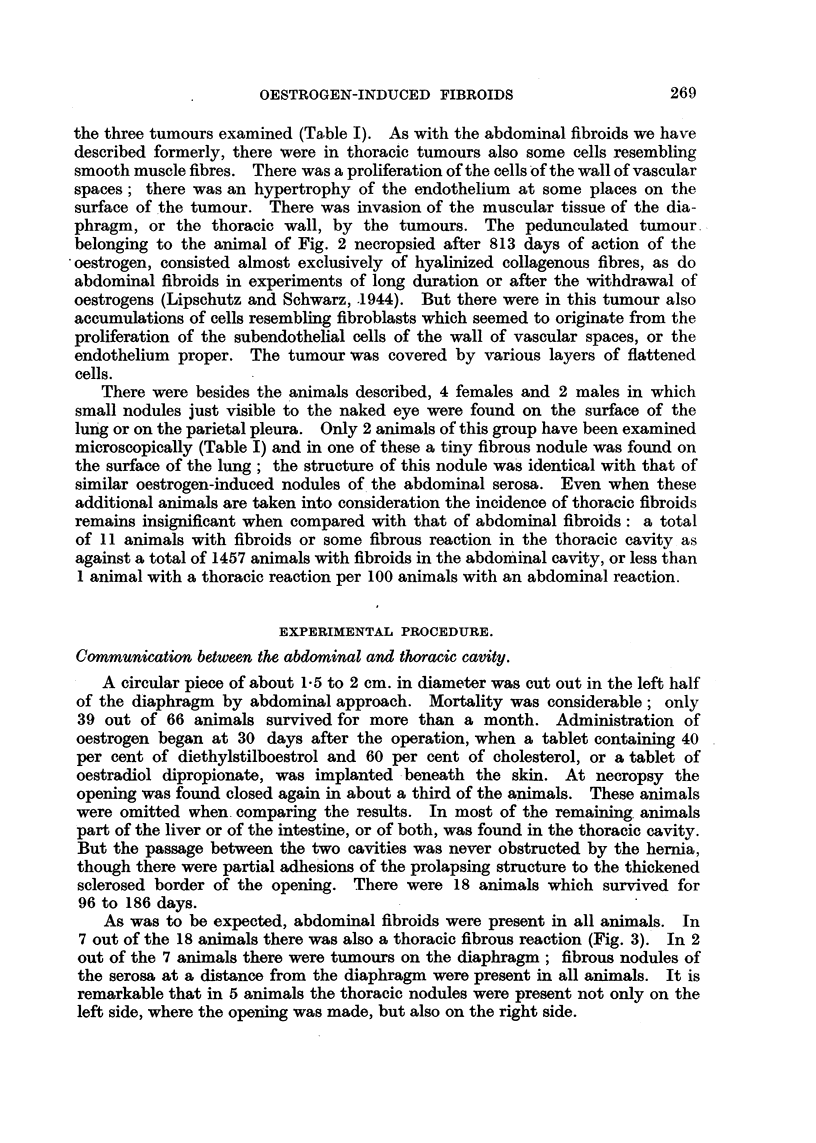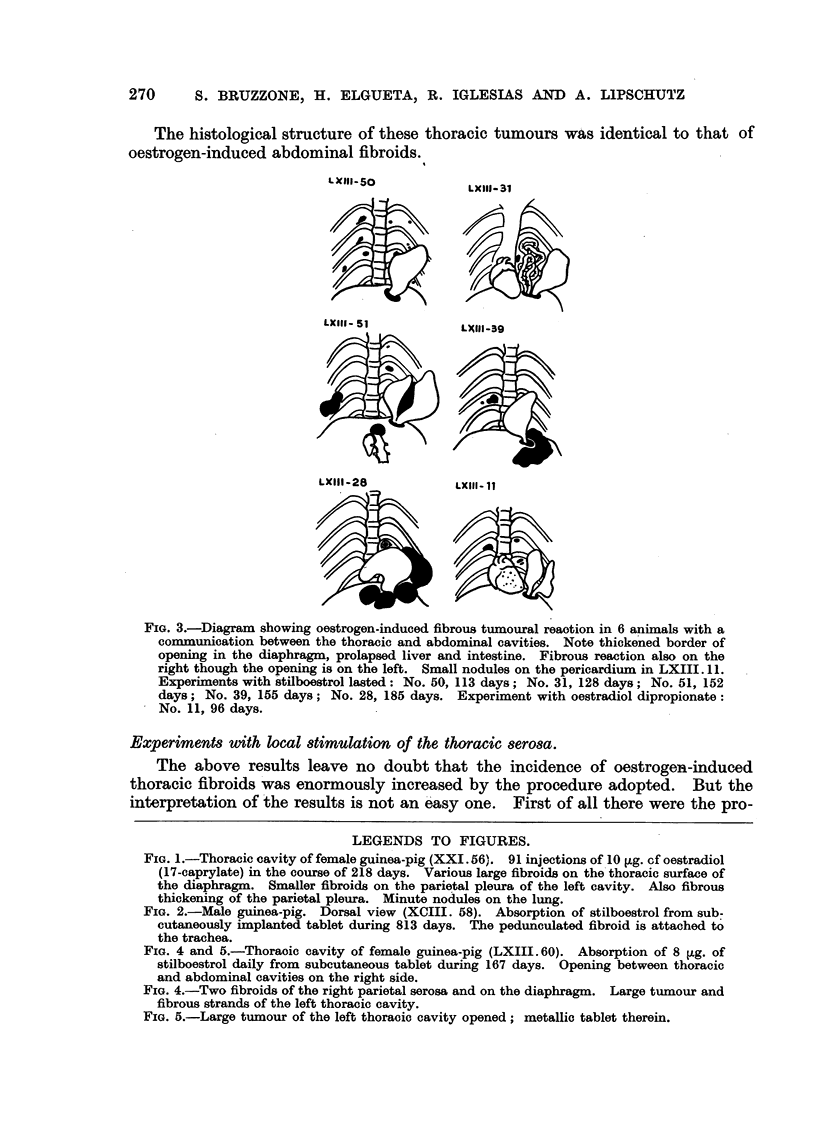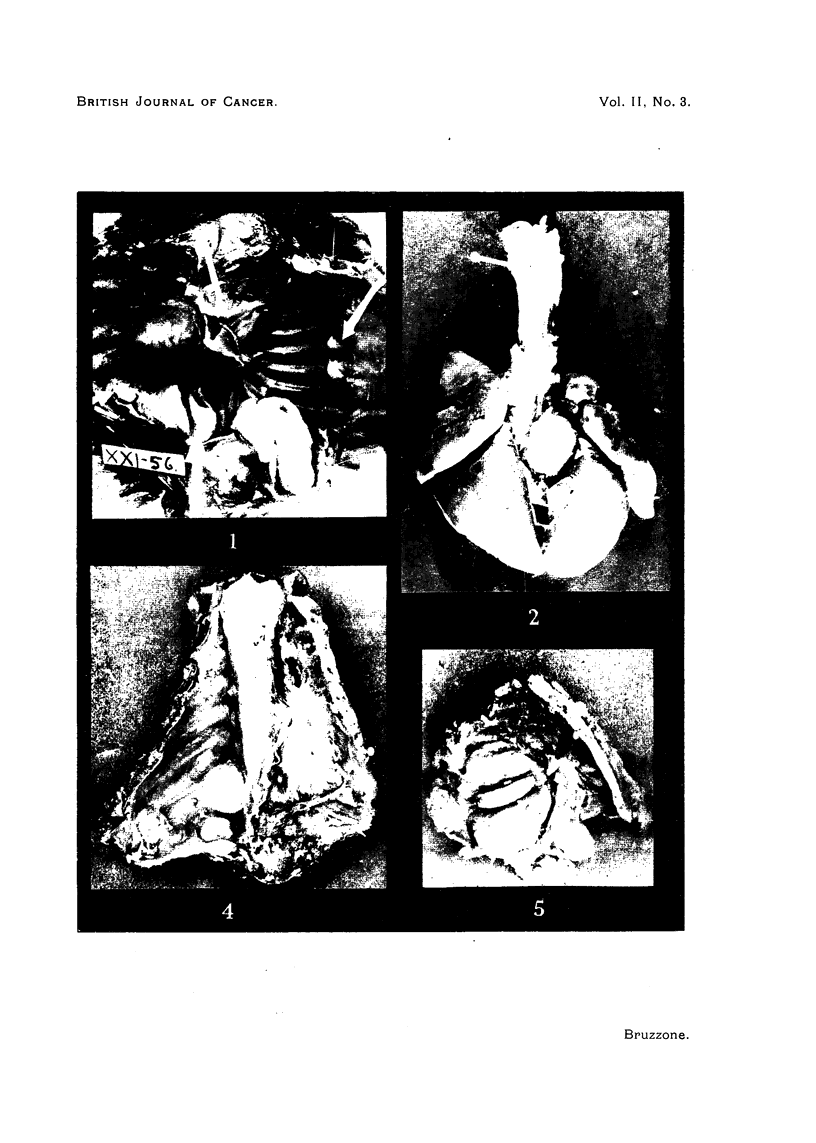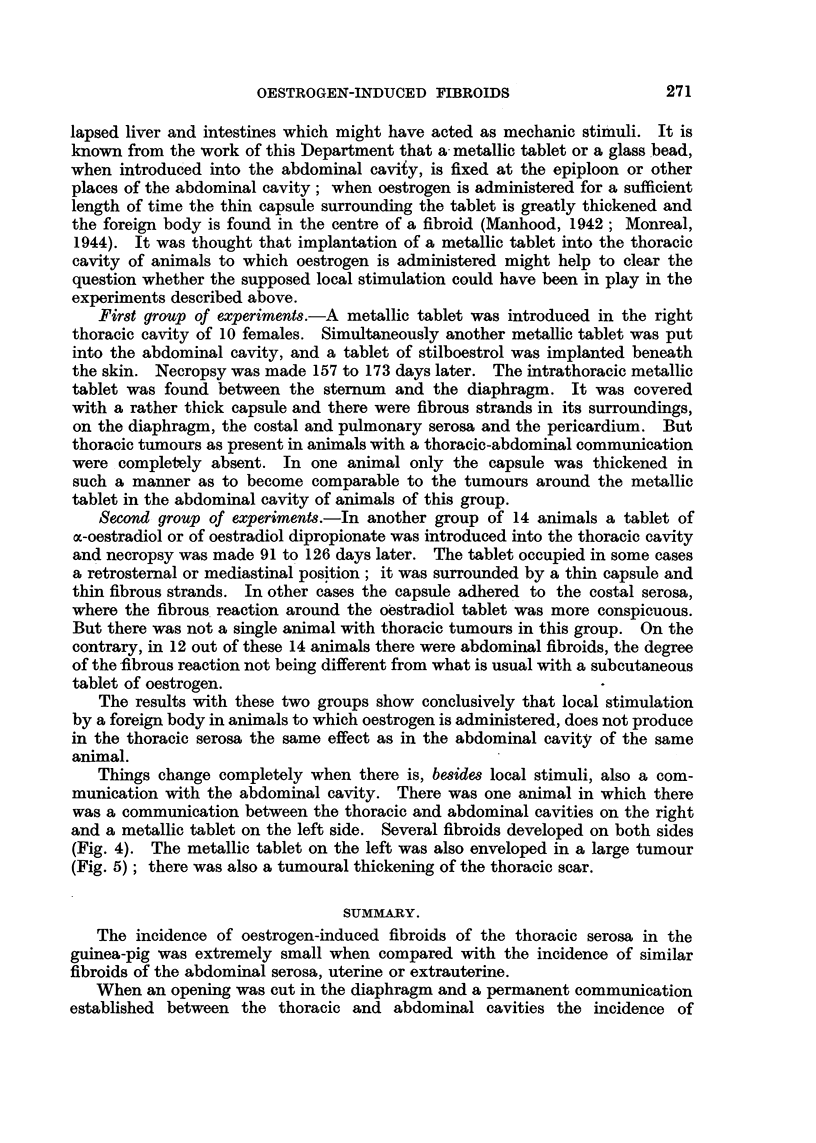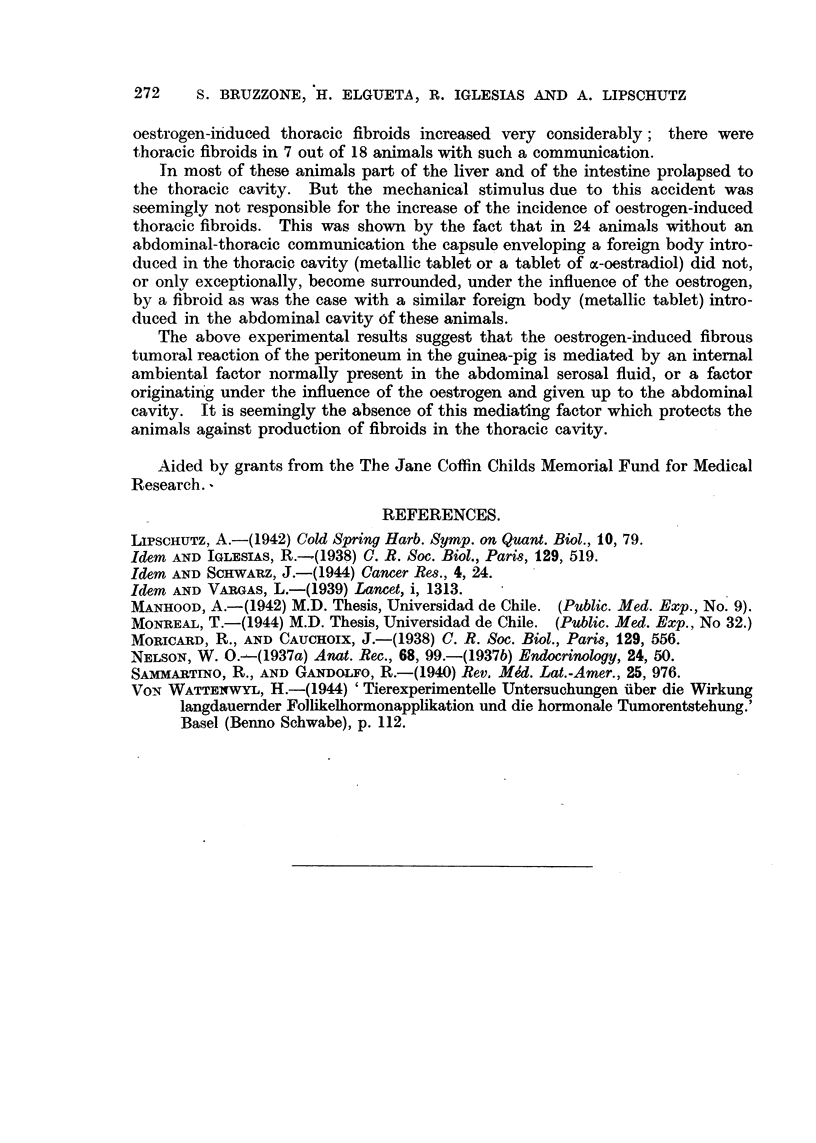# Oestrogen-induced Fibroids of the Thoracic Serosa*

**DOI:** 10.1038/bjc.1948.31

**Published:** 1948-09

**Authors:** S. Bruzzone, H. Elgueta, R. Iglesias, A. Lipschutz

## Abstract

**Images:**


### OESTROGEN-INDUCED FIBROIDS OF THE THORACIC SEROSA.*

S. BRUZZONE, H. ELGUETA, R. IGLESIAS AND A. LIPSCHUTZ.

*From the Department of Experimental Medicine, National Health Service, Santiago de Chile.*

Received for publication July 8, 1948.

UTERINE fibroids have been induced in the guinea-pig by the prolonged administration of oestrogen (Nelson, 1937*a*, 1937*b*; Moricard and Cauchoix, 1938). This finding acquired a more general interest from the point of view of the tumorigenic action of oestrogen in the body when it was discovered that fibroids can be elicited in this species not only on the uterus but on multiple spots of the abdominal serosa (Lipschutz and Iglesias, 1938). It was found that the incidence of these abdominal fibroids can reach up to about 80 per cent or more in female guinea-pigs to which oestrogen is administered continuously for three months (Lipschutz, 1942; Lipschutz and Vargas, 1939). Argentine workers found fibroids on the thoracic side also of the diaphragm in 2 out of 30 animals (Sammartino and Gandolfo, 1940). On the contrary, thoracic fibroids were extremely rare in our own work; it was the same in the extensive work of Von Wattenwyl (1944) with oestrogen-induced abdominal fibroids.

The question arises whether the difference in behaviour of the abdominal and thoracic serosa towards oestrogen is due to a genetically fixed differential reactivity of the endothelial or subendothelial cells of the two serosal covers, or to an internal "ambiental" factor as represented by the abdominal serosal fluid. Results with the experimental proof of the second of these possibilities are given in the present paper.

The above question seems all the more important on account of the following finding. In the male guinea-pig the inguinal channel remains open throughout

* Part of this material has been used by H. Elgueta for his Thesis of M.D. in 1944, for which the Award of the "Liga Nacional del Cáncer" has been received.

the whole life, and when oestrogen is administered, small fibrous nodules appear not only on the abdominal serosa but also on the surface of the testicle.

*Incidence of oestrogen-induced thoracic fibroids.*

Thoracic tumours were found in this Department in 3 out of 1310 females, and in 2 out of 147 males, in which abdominal fibroids were induced by natural or synthetic oestrogens. These findings are summarized in Table I.

TABLE I.—3 *Females and* 2 *Male Guinea-pigs with Oestrogen-induced Thoracic Fibroids, out of* 1310 *Females and* 147 *Males with Abdominal Fibroids. Two Additional Animals with Minor Serosal Reactions.*

| Group. | Duration of treatment (days). | Oestrogen used. | Localization of thoracic tumours. | Histological description. |
|---|---|---|---|---|
| Castr. females : | | | | |
| LXXII.40 | 96 | Oestradiol subcutaneous tablet | Parieto-diaphragmatic | Fibroid with advanced sclerosis; patch of oedematous tissue; probably smooth muscle fibres; proliferation of vascular endothelium; invasion of diaphragm; hypertrophic endothelium on the surface of the tumour. |
| CXXXI.28 | 99 | Oestradiol, subcutaneous tablet | Diaphragmatic | Thick fibrous strands invading the muscular part of the diaphragm; thickening of diaphragmatic serosa. |
| XXI.56 | 218 | Oestradiol - 17 - capryl., injections | Diaphragmatic, parietal and pulmonary (Fig. 1) | Typical fibroid but advanced sclerosis; hyperplastic endothelium of vascular spaces. |
| Castr. males : | | | | |
| LIX.73 | 279 | Oestradiol dipropionate, subcutaneous tablet | Pulmonary | Not examined. |
| XCIII.58 | 813 | Diethylstilboestrol, subcutaneous tablet | Pedunculated tumour, fixed to the trachea (Fig. 2) | Sclerosed tissue ; probably proliferation of vascular endothelium. |
| XCIII.34 | 562 | Diethylstilboestrol, subcutaneous tablet | Pulmonary | Sclerotic nodule ; maximum diameter, about 0 2 mm. |
| XCIII.69 | 734 | Ditto. | ,, | Small cyst of pulmonary pleura ; thick fibrous wall. |

In 2 of the 3 females the tumours were present both on the diaphragm and the thoracic wall (Fig. 1). The parietal tumours are sometimes found at a considerable distance from, and in no connection with, the diaphragm. A tumour in the male was attached to the trachea (Fig. 2). The histological structure of the thoracic tumours was coincident with that of oestrogen-induced abdominal fibroids. The peripheral belt of fibroblasts originating most probably from subendothelial cells of the serosa and so characteristic of the oestrogen-induced abdominal fibroid (Lipschutz, 1942) was present in a diaphragmatic tumour belonging to the animal of Fig. 1. But collagenous fibres prevailed in the three tumours examined (Table I). As with the abdominal fibroids we have described formerly, there were in thoracic tumours also some cells resembling smooth muscle fibres. There was a proliferation of the cells of the wall of vascular spaces; there was an hypertrophy of the endothelium at some places on the surface of the tumour. There was invasion of the muscular tissue of the diaphragm, or the thoracic wall, by the tumours. The pedunculated tumour belonging to the animal of Fig. 2 necropsied after 813 days of action of the oestrogen, consisted almost exclusively of hyalinized collagenous fibres, as do abdominal fibroids in experiments of long duration or after the withdrawal of oestrogens (Lipschutz and Schwarz, 1944). But there were in this tumour also accumulations of cells resembling fibroblasts which seemed to originate from the proliferation of the subendothelial cells of the wall of vascular spaces, or the endothelium proper. The tumour was covered by various layers of flattened cells.

There were besides the animals described, 4 females and 2 males in which small nodules just visible to the naked eye were found on the surface of the lung or on the parietal pleura. Only 2 animals of this group have been examined microscopically (Table I) and in one of these a tiny fibrous nodule was found on the surface of the lung; the structure of this nodule was identical with that of similar oestrogen-induced nodules of the abdominal serosa. Even when these additional animals are taken into consideration the incidence of thoracic fibroids remains insignificant when compared with that of abdominal fibroids: a total of 11 animals with fibroids or some fibrous reaction in the thoracic cavity as against a total of 1457 animals with fibroids in the abdominal cavity, or less than 1 animal with a thoracic reaction per 100 animals with an abdominal reaction.

### EXPERIMENTAL PROCEDURE.

### *Communication between the abdominal and thoracic cavity.*

A circular piece of about 1·5 to 2 cm. in diameter was cut out in the left half of the diaphragm by abdominal approach. Mortality was considerable; only 39 out of 66 animals survived for more than a month. Administration of oestrogen began at 30 days after the operation, when a tablet containing 40 per cent of diethylstilboestrol and 60 per cent of cholesterol, or a tablet of oestradiol dipropionate, was implanted beneath the skin. At necropsy the opening was found closed again in about a third of the animals. These animals were omitted when comparing the results. In most of the remaining animals part of the liver or of the intestine, or of both, was found in the thoracic cavity. But the passage between the two cavities was never obstructed by the hernia, though there were partial adhesions of the prolapsing structure to the thickened sclerosed border of the opening. There were 18 animals which survived for 96 to 186 days.

As was to be expected, abdominal fibroids were present in all animals. In 7 out of the 18 animals there was also a thoracic fibrous reaction (Fig. 3). In 2 out of the 7 animals there were tumours on the diaphragm; fibrous nodules of the serosa at a distance from the diaphragm were present in all animals. It is remarkable that in 5 animals the thoracic nodules were present not only on the left side, where the opening was made, but also on the right side.

The histological structure of these thoracic tumours was identical to that of oestrogen-induced abdominal fibroids.

FIG. 3.—Diagram showing oestrogen-induced fibrous tumoural reaction in 6 animals with a communication between the thoracic and abdominal cavities. Note thickened border of opening in the diaphragm, prolapsed liver and intestine. Fibrous reaction also on the right though the opening is on the left. Small nodules on the pericardium in LXIII.11. Experiments with stilboestrol lasted: No. 50, 113 days; No. 31, 128 days; No. 51, 152 days; No. 39, 155 days; No. 28, 185 days. Experiment with oestradiol dipropionate: No. 11, 96 days.

*Experiments with local stimulation of the thoracic serosa.*

The above results leave no doubt that the incidence of oestrogen-induced thoracic fibroids was enormously increased by the procedure adopted. But the interpretation of the results is not an easy one. First of all there were the pro-

---

LEGENDS TO FIGURES.

FIG. 1.—Thoracic cavity of female guinea-pig (XXI.56). 91 injections of 10 μg. of oestradiol (17-caprylate) in the course of 218 days. Various large fibroids on the thoracic surface of the diaphragm. Smaller fibroids on the parietal pleura of the left cavity. Also fibrous thickening of the parietal pleura. Minute nodules on the lung.

FIG. 2.—Male guinea-pig. Dorsal view (XCIII. 58). Absorption of stilboestrol from subcutaneously implanted tablet during 813 days. The pedunculated fibroid is attached to the trachea.

FIG. 4 and 5.—Thoracic cavity of female guinea-pig (LXIII.60). Absorption of 8 μg. of stilboestrol daily from subcutaneous tablet during 167 days. Opening between thoracic and abdominal cavities on the right side.

FIG. 4.—Two fibroids of the right parietal serosa and on the diaphragm. Large tumour and fibrous strands of the left thoracic cavity.

FIG. 5.—Large tumour of the left thoracic cavity opened; metallic tablet therein.

1

2

4

5

lapsed liver and intestines which might have acted as mechanic stimuli. It is known from the work of this Department that a metallic tablet or a glass bead, when introduced into the abdominal cavity, is fixed at the epiploon or other places of the abdominal cavity ; when oestrogen is administered for a sufficient length of time the thin capsule surrounding the tablet is greatly thickened and the foreign body is found in the centre of a fibroid (Manhood, 1942 ; Monreal, 1944). It was thought that implantation of a metallic tablet into the thoracic cavity of animals to which oestrogen is administered might help to clear the question whether the supposed local stimulation could have been in play in the experiments described above.

*First group of experiments.*—A metallic tablet was introduced in the right thoracic cavity of 10 females. Simultaneously another metallic tablet was put into the abdominal cavity, and a tablet of stilboestrol was implanted beneath the skin. Necropsy was made 157 to 173 days later. The intrathoracic metallic tablet was found between the sternum and the diaphragm. It was covered with a rather thick capsule and there were fibrous strands in its surroundings, on the diaphragm, the costal and pulmonary serosa and the pericardium. But thoracic tumours as present in animals with a thoracic-abdominal communication were completely absent. In one animal only the capsule was thickened in such a manner as to become comparable to the tumours around the metallic tablet in the abdominal cavity of animals of this group.

*Second group of experiments.*—In another group of 14 animals a tablet of α-oestradiol or of oestradiol dipropionate was introduced into the thoracic cavity and necropsy was made 91 to 126 days later. The tablet occupied in some cases a retrosternal or mediastinal position ; it was surrounded by a thin capsule and thin fibrous strands. In other cases the capsule adhered to the costal serosa, where the fibrous reaction around the oestradiol tablet was more conspicuous. But there was not a single animal with thoracic tumours in this group. On the contrary, in 12 out of these 14 animals there were abdominal fibroids, the degree of the fibrous reaction not being different from what is usual with a subcutaneous tablet of oestrogen.

The results with these two groups show conclusively that local stimulation by a foreign body in animals to which oestrogen is administered, does not produce in the thoracic serosa the same effect as in the abdominal cavity of the same animal.

Things change completely when there is, *besides* local stimuli, also a communication with the abdominal cavity. There was one animal in which there was a communication between the thoracic and abdominal cavities on the right and a metallic tablet on the left side. Several fibroids developed on both sides (Fig. 4). The metallic tablet on the left was also enveloped in a large tumour (Fig. 5) ; there was also a tumoural thickening of the thoracic scar.

### SUMMARY.

The incidence of oestrogen-induced fibroids of the thoracic serosa in the guinea-pig was extremely small when compared with the incidence of similar fibroids of the abdominal serosa, uterine or extrauterine.

When an opening was cut in the diaphragm and a permanent communication established between the thoracic and abdominal cavities the incidence of oestrogen-induced thoracic fibroids increased very considerably; there were thoracic fibroids in 7 out of 18 animals with such a communication.

In most of these animals part of the liver and of the intestine prolapsed to the thoracic cavity. But the mechanical stimulus due to this accident was seemingly not responsible for the increase of the incidence of oestrogen-induced thoracic fibroids. This was shown by the fact that in 24 animals without an abdominal-thoracic communication the capsule enveloping a foreign body introduced in the thoracic cavity (metallic tablet or a tablet of α-oestradiol) did not, or only exceptionally, become surrounded, under the influence of the oestrogen, by a fibroid as was the case with a similar foreign body (metallic tablet) introduced in the abdominal cavity of these animals.

The above experimental results suggest that the oestrogen-induced fibrous tumoral reaction of the peritoneum in the guinea-pig is mediated by an internal ambiental factor normally present in the abdominal serosal fluid, or a factor originating under the influence of the oestrogen and given up to the abdominal cavity. It is seemingly the absence of this mediating factor which protects the animals against production of fibroids in the thoracic cavity.

Aided by grants from the The Jane Coffin Childs Memorial Fund for Medical Research.

### REFERENCES.

LIPSCHUTZ, A.—(1942) *Cold Spring Harb. Symp. on Quant. Biol.*, **10**, 79.
*Idem* AND IGLESIAS, R.—(1938) *C. R. Soc. Biol., Paris*, **129**, 519.
*Idem* AND SCHWARZ, J.—(1944) *Cancer Res.*, **4**, 24.
*Idem* AND VARGAS, L.—(1939) *Lancet*, i, 1313.
MANHOOD, A.—(1942) M.D. Thesis, Universidad de Chile. (*Public. Med. Exp.*, No. 9).
MONREAL, T.—(1944) M.D. Thesis, Universidad de Chile. (*Public. Med. Exp.*, No 32.)
MORICARD, R., AND CAUCHOIX, J.—(1938) *C. R. Soc. Biol., Paris*, **129**, 556.
NELSON, W. O.—(1937*a*) *Anat. Rec.*, **68**, 99.—(1937*b*) *Endocrinology*, **24**, 50.
SAMMARTINO, R., AND GANDOLFO, R.—(1940) *Rev. Méd. Lat.-Amer.*, **25**, 976.
VON WATTENWYL, H.—(1944) 'Tierexperimentelle Untersuchungen über die Wirkung langdauernder Follikelhormonapplikation und die hormonale Tumorentstehung.' Basel (Benno Schwabe), p. 112.